# Supplementary material for: Deciphering the complex role of thrombospondin-1 in glioblastoma development
Source: Nat Commun. 2019 Mar 8;10:1146. doi: 10.1038/s41467-019-08480-y (PMC6408502; doi:10.1038/s41467-019-08480-y)
Supplement: Supplementary file 5 — Reporting Summary [file 41467_2019_8480_MOESM5_ESM.pdf]

nature research

Corresponding author(s): Pr. Andreas Bikfalvi and Dr. Thomas Daubon

Last updated by author(s): 2018/12/08

Reporting Summary

Nature Research wishes to improve the reproducibility of the work that we publish. This form provides structure for consistency and transparency in reporting. For further information on Nature Research policies, see Authors & Referees and the Editorial Policy Checklist.

Statistics

For all statistical analyses, confirm that the following items are present in the figure legend, table legend, main text, or Methods section.

n/a	Confirmed	
☐	☒	The exact sample size (*n*) for each experimental group/condition, given as a discrete number and unit of measurement
☐	☒	A statement on whether measurements were taken from distinct samples or whether the same sample was measured repeatedly
☐	☒	The statistical test(s) used AND whether they are one- or two-sided<br>*Only common tests should be described solely by name; describe more complex techniques in the Methods section.*
☒	☐	A description of all covariates tested
☐	☒	A description of any assumptions or corrections, such as tests of normality and adjustment for multiple comparisons
☐	☒	A full description of the statistical parameters including central tendency (e.g. means) or other basic estimates (e.g. regression coefficient) AND variation (e.g. standard deviation) or associated estimates of uncertainty (e.g. confidence intervals)
☐	☒	For null hypothesis testing, the test statistic (e.g. *F*, *t*, *r*) with confidence intervals, effect sizes, degrees of freedom and *P* value noted<br>*Give P values as exact values whenever suitable.*
☒	☐	For Bayesian analysis, information on the choice of priors and Markov chain Monte Carlo settings
☒	☐	For hierarchical and complex designs, identification of the appropriate level for tests and full reporting of outcomes
☐	☒	Estimates of effect sizes (e.g. Cohen's *d*, Pearson's *r*), indicating how they were calculated

Our web collection on statistics for biologists contains articles on many of the points above.

Software and code

Policy information about availability of computer code

Data collection	For generating graphs or curves, GraphPad Prism 6 software was used. Fiji (ImageJ) was used for changing image colors.
Data analysis	GraphPad Prism 6 software was used for generating all data and statistics.

For manuscripts utilizing custom algorithms or software that are central to the research but not yet described in published literature, software must be made available to editors/reviewers. We strongly encourage code deposition in a community repository (e.g. GitHub). See the Nature Research guidelines for submitting code & software for further information.

Data

Policy information about availability of data

All manuscripts must include a data availability statement. This statement should provide the following information, where applicable:

- Accession codes, unique identifiers, or web links for publicly available datasets
- A list of figures that have associated raw data
- A description of any restrictions on data availability

All data are available within the Article and Supplementary Files, or available from the authors upon request. Transcriptomics data is available from the SRA: PRJNA320047 or the European Nucleotide Archive: PRJEB23786.

Field-specific reporting

Please select the one below that is the best fit for your research. If you are not sure, read the appropriate sections before making your selection.

☒ Life sciences ☐ Behavioural & social sciences ☐ Ecological, evolutionary & environmental sciences

Life sciences study design

All studies must disclose on these points even when the disclosure is negative.

Sample size	For in vitro experiments, we took at least 8 replicated per experiment and then at least 3 to 4 independent experiments were performed. Some of the critical experiments were done by two independent investigators (double blind by Dr. Thomas Daubon et Céline Léon). We initially used R software for determining a power of 0.95. For animal experiment (survival analysis), 7 to 8 animals were used per group, as determined by R software, for getting a power of 0.95.
Data exclusions	No data were excluded from the experiments, as a sufficient number of replicates was included in each independent experiment.
Replication	Statistical test were assessed to compare the means of each experiment with the 3 or 4 independent experiments. Anova test was used to compare multi-sample experiments and student test for comparing 2 conditions.
Randomization	After mouse implantation, the animals were randomly distributed into cages for avoiding littermate variation, for the further treatments.
Blinding	Experiments from Figures 2, 3A, 3D, 4C, 4D, 4E, 5A, 6B, 6C have been done by two independent experimentators.

Reporting for specific materials, systems and methods

We require information from authors about some types of materials, experimental systems and methods used in many studies. Here, indicate whether each material, system or method listed is relevant to your study. If you are not sure if a list item applies to your research, read the appropriate section before selecting a response.

Materials & experimental systems

n/a	Involved in the study	
☐	☒	Antibodies
☐	☒	Eukaryotic cell lines
☒	☐	Palaeontology
☐	☒	Animals and other organisms
☒	☐	Human research participants
☐	☒	Clinical data

Methods

n/a	Involved in the study	
☒	☐	ChIP-seq
☒	☐	Flow cytometry
☒	☐	MRI-based neuroimaging

Antibodies

Antibodies used

Antibody Manufacturer Cat. No. Working Conc/Dilution Application Host
AKT (11E7) Cell Signaling 4685 1/10000 WB Rabbit
CAIX (Carboxy Anhydrase IX) Abcam ab15086 1/500 IF Rabbit
CD31 BD Pharmingen 553370 1/100 IF Rat
CD47 BD Pharmingen 556044 1/500 WB Mouse
Cortactin Millipore 05-180 1/500 IF Mouse
DAPI Fisher D1306 1/106 IF
Endoglin (CD105) BD Pharmingen 550546 1/50 IF Rat
F-actin (I-19) Santa Cruz sc-1616 1/1000 IF Goat
Fibronectin Abcam ab154211 1/1000 WB Mouse
Fibronectin Abcam ab23750 1/600 IF Rabbit
IRE1 α Santa Cruz sc-20790 1/1000 WB Rabbit
Nestin (clone 10C2) Millipore MAB5326 1/500 IF Mouse
pAKT (Ser473) Cell Signaling 9271 1/1000 WB Rabbit
pERK Cell Signaling 9101 1/1000 WB Rabbit
Phalloidin Rhodamine Molecular Probes R415 1/100 IF
Psmad1-5 (S463/465) (41D10) Cell Signaling 9516 1/500 WB Rabbit
Psmad2 (S465/467)/Smad3 (S423/425) (D27F4) Cell Signaling 8828 1/100 IF Rabbit
Psmad2 (Ser465/467) Cell Signaling 3108 1/500 WB Rabbit
Psmad3 Abcam ab52903 1/100 IF Rabbit
Psmad3 (S423/425) (C25A9) Cell Signaling 9520 1/500 WB Rabbit
Smad1 (D59D7) Cell Signaling 6944 1/500 WB Rabbit
Smad2 (D43B4) Cell Signaling 5339 1/500 WB Rabbit
Smad3 (C67H9) Cell Signaling 9523 1/500 WB Rabbit
Smad4 Cell Signaling 9515 1/500 IF Rabbit
TGFb1 Abcam ab9758 1/200 WB Rabbit
THBS1 Santa Cruz 59886 1/200 HIC Mouse
THBS1 Home-made from JJ.Feige 1/500 IF Rabbit
THBS1 (A6.1) Thermo-Scientific MA5-13398 1/500 WB Mouse
Tubulin Sigma T5168 1/5000 WB Mouse

	Vimentin Abcam ab16700 1/200 IF Rabbit<br>Vinculin Sigma V9131 1/500 WB Mouse
Validation	All commercial antibodies were validated by both manufacturers and independent laboratories. The home-made THBS1 antibodies coming from JJ Feige lab were validated in de Fraipont F et al, Oncogene 2004.

Eukaryotic cell lines

Policy information about cell lines

Cell line source(s)	U87MG cells were pruchased from ATCC.
Authentication	All cells used for phenotypic and functional studies have been further characterized more in detail by cGH array (P3) and by cell authentication (U87) using Promega Powerplex21 Kit (Eurofins, GE).
Mycoplasma contamination	The U87 (ATCC) and P3 glioma cell lines were regularly tested for mycoplasma contamination and were all mycoplasma-free.
Commonly misidentified lines<br>(See ICLAC register)	No misidentified cell line was used in this study.

Animals and other organisms

Policy information about studies involving animals; ARRIVE guidelines recommended for reporting animal research

Laboratory animals	Male RAGy2C-/- mice were housed and treated in the animal facility of Bordeaux University (“Animalerie Mutualisée Bordeaux”).
Wild animals	No wild animal was used in this study.
Field-collected samples	No such sample was used in this study.
Ethics oversight	All animal procedures have been done according to the institutional guidelines and approved by the local ethics committee (agreement number: 4611).

Note that full information on the approval of the study protocol must also be provided in the manuscript.

Clinical data

Policy information about clinical studies

All manuscripts should comply with the ICMJE guidelines for publication of clinical research and a completed CONSORT checklist must be included with all submissions.

Clinical trial registration	No clinical trial was performed in this study.
Study protocol	No clinical trial was performed in this study.
Data collection	No clinical trial was performed in this study.
Outcomes	No clinical trial was performed in this study.
