# Supplementary material for: Deciphering the complex role of thrombospondin-1 in glioblastoma development
Source: Nat Commun. 2019 Mar 8;10:1146. doi: 10.1038/s41467-019-08480-y (PMC6408502; doi:10.1038/s41467-019-08480-y)
Supplement: Supplementary file 6 — Description of Additional Supplementary Files [file 41467_2019_8480_MOESM6_ESM.docx]

**Title:** Supplementary Data 1
**Description:** Mouse and human genes classified with DESeq2 in P3 tumours. Two-class comparisons between core and invasive samples using the R package DESeq2 were performed with human (sheet 1) and mouse (sheet 2) genes in P3 tumours.

**Title:** Supplementary Data 2
**Description:** Differential expression of genes in P3 tumours Differentially expressed genes were defined by using an upper threshold on the p-value of 0.01 after correction for multiple testing.

**Title:** Supplementary Data 3
**Description:** Gene ontology of human and mouse genes from P3 tumours.
