# Supplementary material for: Deciphering the complex role of thrombospondin-1 in glioblastoma development
Source: Nat Commun. 2019 Mar 8;10:1146. doi: 10.1038/s41467-019-08480-y (PMC6408502; doi:10.1038/s41467-019-08480-y)
Supplement: Supplementary file 1 — Supplementary information [file 41467_2019_8480_MOESM1_ESM.pdf]

SUPPLEMENTARY INFORMATION

Deciphering the complex role of thrombospondin-1 in glioblastoma development

Daubon et al.

A

GENE_SYMBOL	fold G4 vs G2 (log2)	G2 mean	G3 mean	G4 mean	Absolute t value (G2 vs G4)	Raw p value (G2 vs G4)	Adj p value (G2 vs G4)
THBS1	4.82	6.32	6.77	8.59	5.77	1.69E-07	2.39E-06
TGFB1	1.53	9.13	9.39	9.75	4.21	6.12E-05	4.05E-04

B

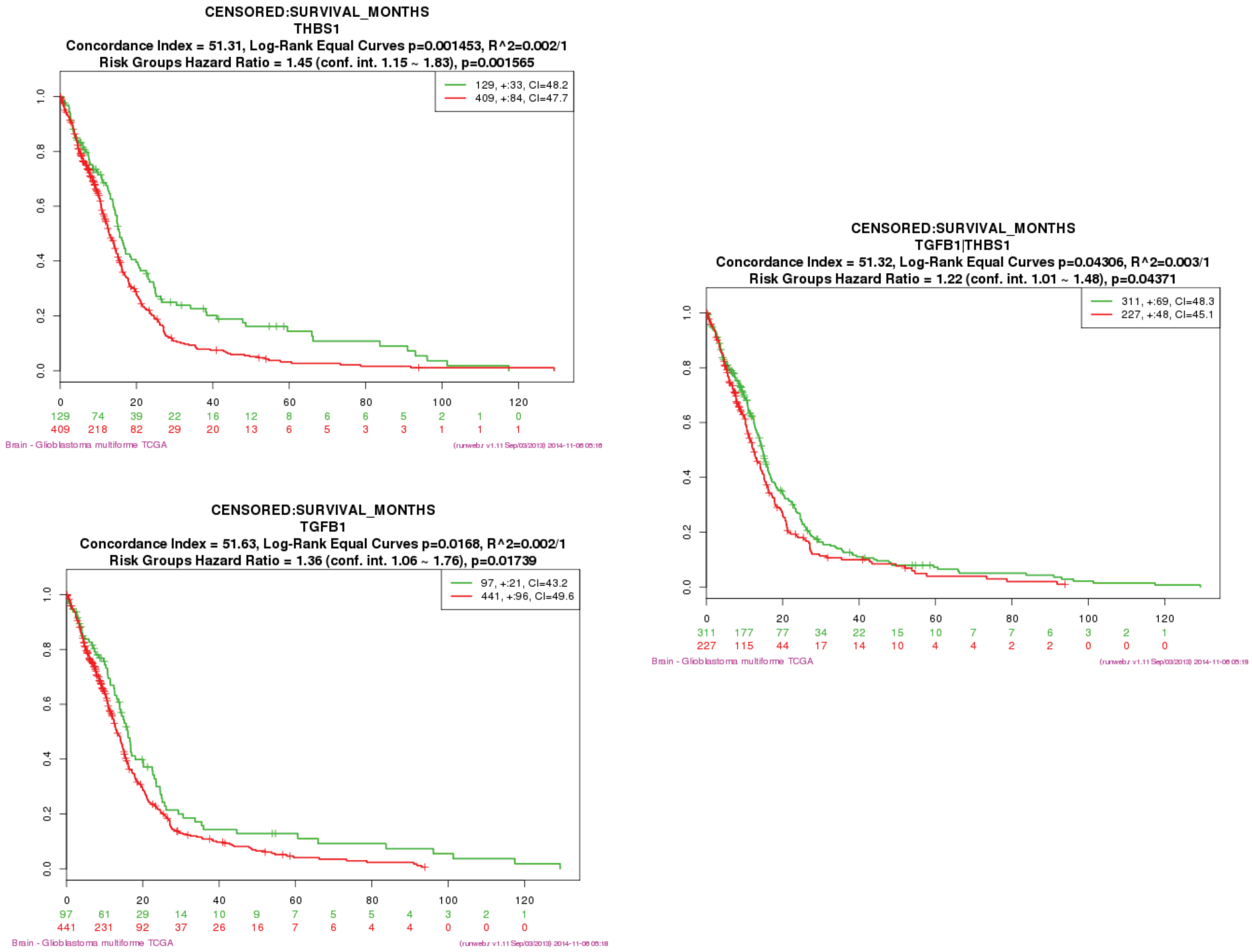

<u>Supplementary figure 1</u>: Survival analysis and expressions of *THBS1* and *TGFB1* genes in GBM

(A) *In silico* analysis of TBHS1 and TGFβ1 expressions in gliomas of various grades (data extracted from Sun et al.[1]). The fold changes are represented in log2 scale. This gives a log2 fold change of 8.59 minus 6.32 = 2.27. To convert this to a linear fold change we calculate 2 to the power of 2.27 (2^2.27 = 4.82). THBS1 appears highly upregulated in grade IV gliomas (G4) compared to grade II (G2). TGFβ1 is slightly upregulated in grade IV compared to grade II.

(B) Survival analysis using THBS1 (upper panel), TGFB1 (central panel) or combined THBS1/TGFB1 (lower panel) expression in glioma grade IV patients. Significant correlation between patient survival and THBS1 expression is observed. TGFB1 or combined analysis did not represent a good predictor for patient survival.

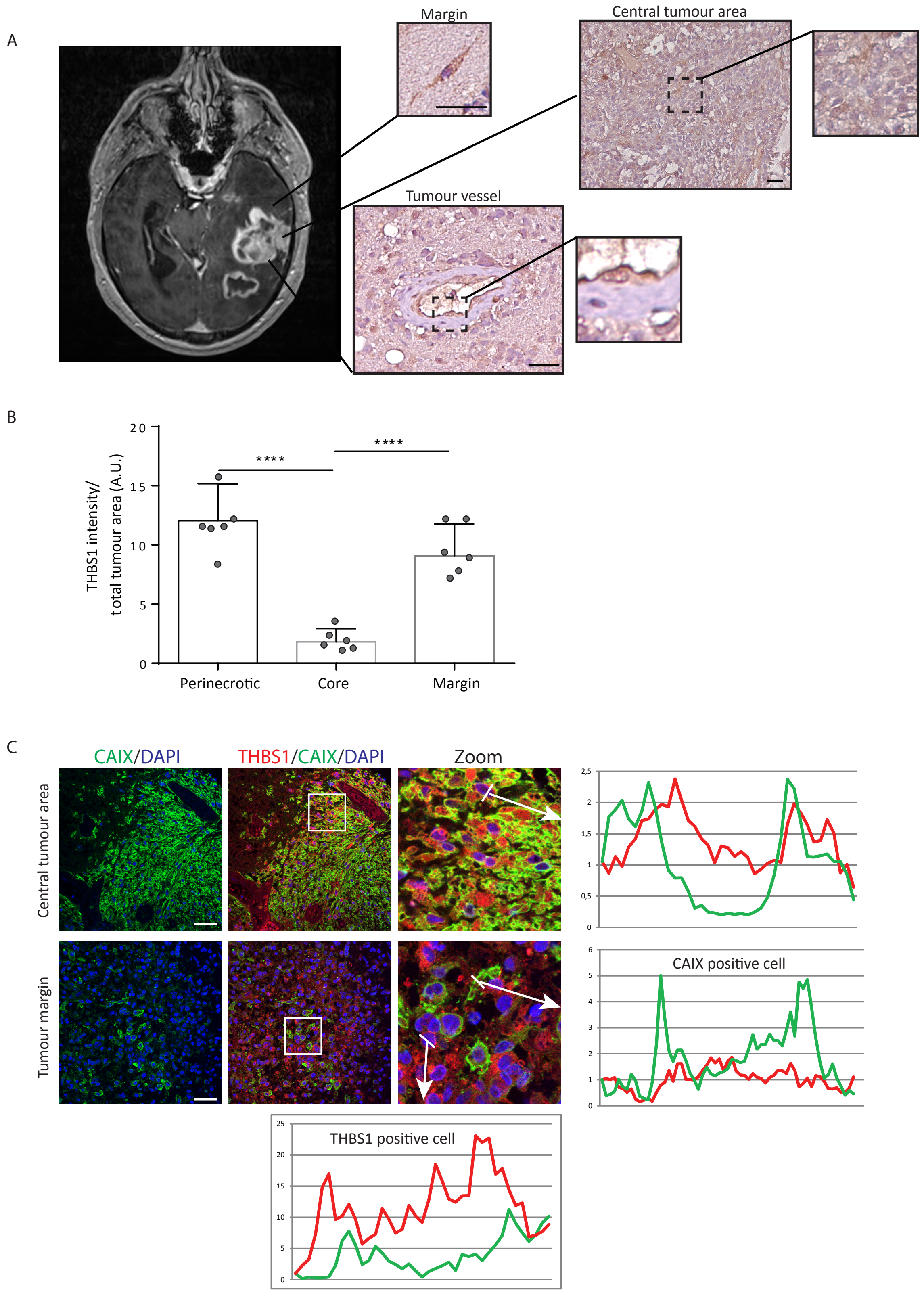

<u>Supplementary figure 2</u>: THBS1 is expressed in hypoxic areas, tumour vessels and tumour margins

(A) Immunohistological analysis of patients with glioblastoma. The left panel represents an MRI image (T2) and the adjacent panels depict immunostaining of THBS1 in the margin, in the intermediate area and in the central hypoxic area. THBS1 is expressed in invasive cells and in perinecrotic areas. Positive immunoreactivity is also present in tumour blood vessels. Scale bars: 50 µm.

(B) THBS1 signal intensity was analysed on samples from 6 patients by using IHC profiler from Fiji software (molecular profiles of 6 patients in Supplementary Table 1). The results are expressed as means ± s.d. **** P< 0.001 (ANOVA).

(C) Immunostaining of THBS1 (red) and Carboxy Anhydrase IX (CAIX in green) in the margin, in the intermediate area and in the central tumour area. DAPI was used for nuclear staining (blue). Scale bars: 50 µm. The graphs represent plot analysis of CAIX (green) and THBS1 (red) expressions of single cells, using Fiji software (represented areas marked by white lines in the images).

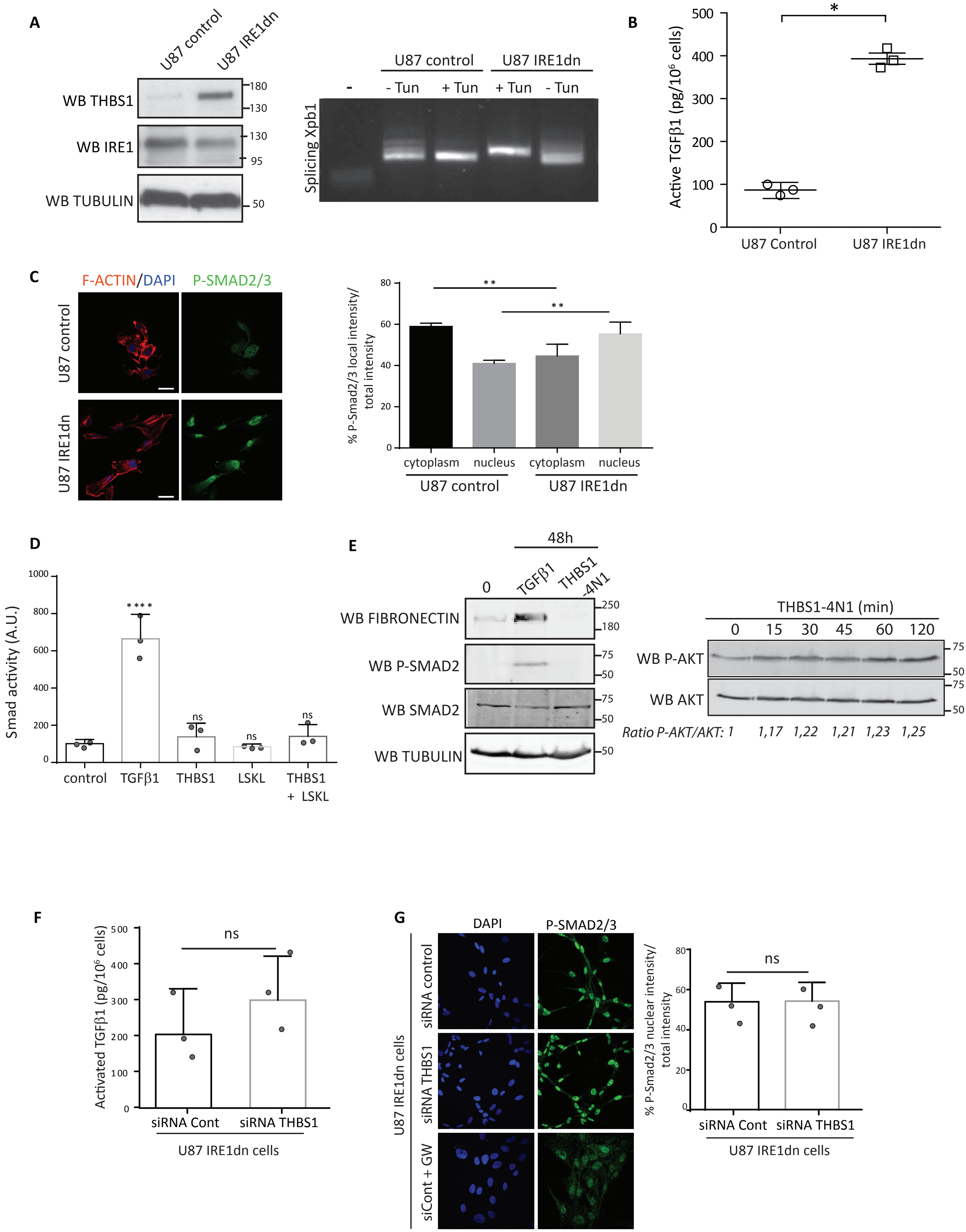
A
U87 control
U87 IRE1dn
WB THBS1
WB IRE1
WB TUBULIN
180
130
130
95
50
U87 control
U87 IRE1dn
-
- Tun
+ Tun
+ Tun
- Tun
Splicing Xpb1
B
Active TGFβ1 (pg/10⁶ cells)
500
400
300
200
100
0
*
U87 Control
U87 IRE1dn
C
F-ACTIN/DAPI
P-SMAD2/3
U87 control
U87 IRE1dn
% P-Smad2/3 local intensity/ total intensity
80
60
40
20
0
**
**
cytoplasm
nucleus
cytoplasm
nucleus
U87 control
U87 IRE1dn
D
Smad activity (A.U.)
1000
800
600
400
200
0
****
ns
ns
ns
control
TGFβ1
THBS1
LSKL
THBS1 + LSKL
E
48h
0
TGFβ1
THBS1 -4N1
WB FIBRONECTIN
WB P-SMAD2
WB SMAD2
WB TUBULIN
250
180
75
50
75
50
50
THBS1-4N1 (min)
0
15
30
45
60
120
WB P-AKT
WB AKT
75
50
75
50
Ratio P-AKT/AKT: 1
1,17
1,22
1,21
1,23
1,25
F
Activated TGFβ1 (pg/10⁶ cells)
500
400
300
200
100
0
ns
siRNA Cont
siRNA THBS1
U87 IRE1dn cells
G
DAPI
P-SMAD2/3
siRNA control
siRNA THBS1
siCont + GW
U87 IRE1dn cells
% P-Smad2/3 nuclear intensity/ total intensity
80
60
40
20
0
ns
siRNA Cont
siRNA THBS1
U87 IRE1dn cells

<u>Supplementary figure 3</u>: THBS1 does not activate TGFβ1 in U87 models

(**A**) Immunoblots of protein extracts from control or IRE1dn U87 cells probed with anti-THBS1, anti-IRE1 or anti-Tubulin antibodies (left panel). For controlling IRE1 status, Xbp1 splicing in both cell lines, treated or not with 10 µg/ml of tunicamycin is shown (right panel).

(**B**) Measurement of active TGFβ1 in supernatants of control or IRE1dn U87 cells. The graph represents the results as means ± s.d. of 3 independent experiments (*p < 0.05 ; Student's t-test).

(**C**) Representative images of control or IRE1dn U87 cells showing nuclear translocation of phospho-Smad2/3 for IRE1dn cells. Scale bars: 10 µm. The graph represents phospho-Smad2/3 localisation in control and IRE1dn U87 cells. The results are expressed as means ± s.d. ** P< 0.01 (ANOVA).

(**D**) A construct harbouring the Smad Responsive Element (SRE) was transfected into U87 cells. Cells were treated with 5 ng/ml of recombinant TGFβ1, 10 µg/ml of purified THBS1, or 10 µg/ml of LSKL peptide. The graph represents the results as means ± s.d. of three independent experiments of fold induction compared to non-treated cells. **** P< 0.001 ; ns, non significant (ANOVA).

(**E**) Immunoblots from protein extracts of U87 cells treated with 5 ng/ml of recombinant TGFβ1 or 10 µg/ml of recombinant THBS1 for 48 hours, and probed for THBS1, P-SMAD2, total SMAD2 or Tubulin (left panel). Immunoblots from protein extracts of U87 cells treated with recombinant THBS1 during 15, 30, 45, 60 or 120 min, and probed for phospho-AKT or total AKT (right panel). Ratios below were calculated from the signal intensities of P-AKT on total AKT.

(**F**) Measurement of active TGFβ1 in supernatants of control or THBS1 siRNA-treated U87 IRE1dn cells. The graph represents the results as means ± s.d. of 3 independent experiments. ns, not significant (Student t-test).

(**G**) Immunostaining with anti-phospho-SMAD2/3 antibody of control or THBS1 siRNA-treated U87 IRE1dn cells. Scale bars: 20 µm. Quantification of nuclear and cytoplasmic P-SMAD2/3 from immunofluorescence images. The results represent the means ± s.d. of 3 independent experiments (n= 90 cells in each experiment) ; ns, not significant (Student t-test). As a control, U87 IRE1 dn cells were treated with TGFβ receptor I inhibitor GW788388 (GW).

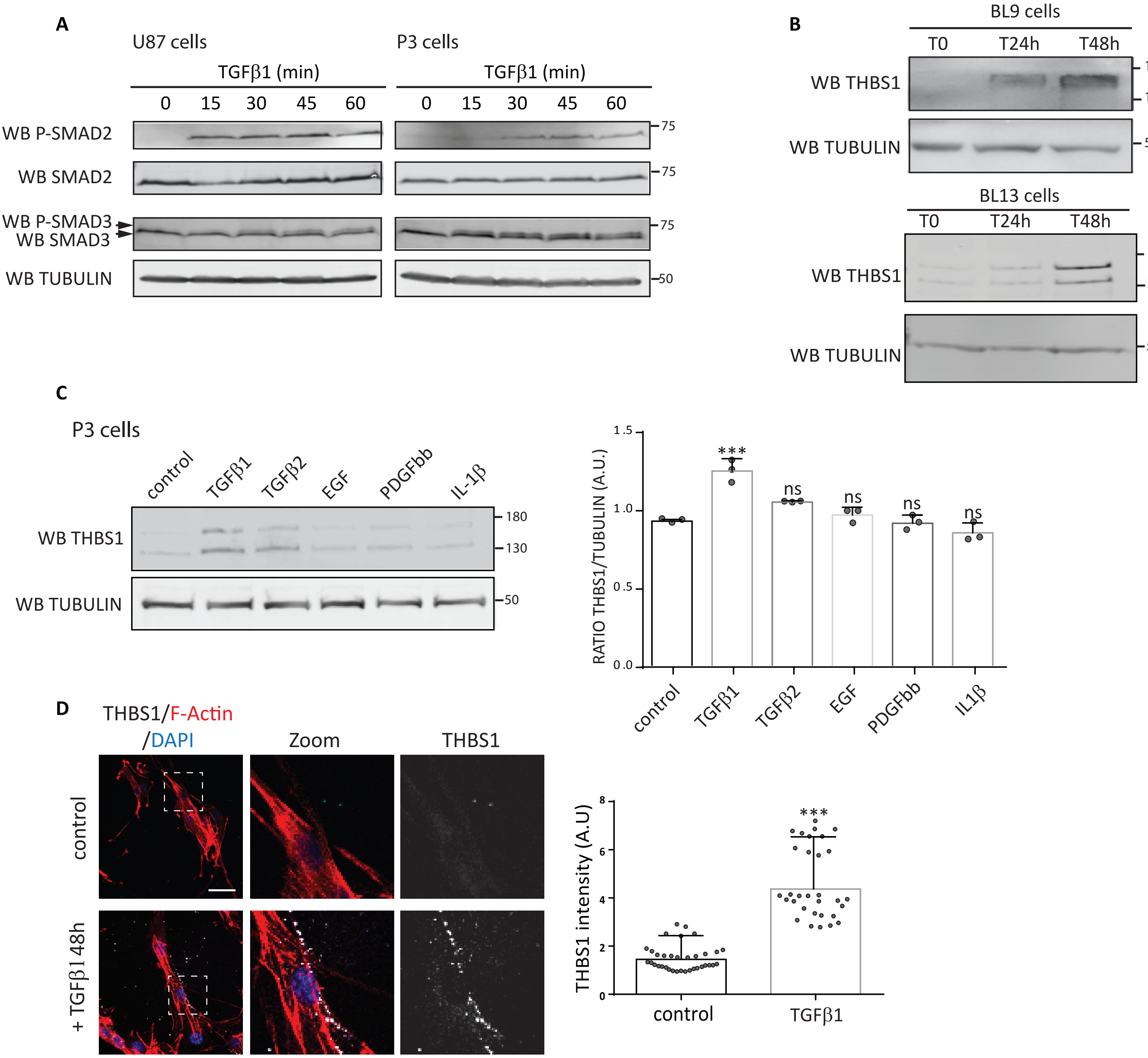

Supplementary figure 4 : TGFβ1 treatment increases THBS1 expression in U87 and patient-derived cells

(**A**) Immunoblots of protein extracts from starved U87 and P3 cells treated with 5 ng/ml of recombinant TGFβ1 for 15, 30, 45 or 60 minutes, and probed with anti P-SMAD2, total SMAD2, total SMAD3 (recognizing both phosphorylated and total forms), or anti-Tubulin antibodies.

(**B**) Immunoblots of protein extracts from patient-derived BL9 and BL13 cells treated with 5 ng/ml of recombinant TGFβ1 for 24 or 48 hours, and probed with anti-THBS1 or anti-Tubulin antibodies.

(**C**) Immunoblots of protein extracts from P3 cells treated with 5 ng/ml of recombinant TGFβ1, 10 ng/ml of recombinant TGFβ2, 20 ng/ml of EGF, 10 ng/ml of PDGF-BB or 20 ng/ml of IL1β, and probed with anti-THBS1 or anti-Tubulin antibodies. The graph on the right represents ratios of THBS1 signal intensity over tubulin intensity. The results are represented as means ± s.d. of three independent experiments. *** P< 0.001 ; ns, non-significant (ANOVA).

(**D**) Immunostaining of TGFβ-treated or untreated U87 cells probed with THBS1 antibody. Representative images of U87 cells treated with 5 ng/ml of recombinant TGFβ1 during 48 hours showing deposits of THBS1. (THBS1, white ; F-actin, red ; and DAPI, blue). THBS1 is detected as little dots along and around cells. Scale bars: 10 µm. The graph below represents quantification of the THBS1 signal from several images (n= 35). *** p < 0.001 (Student t-test).

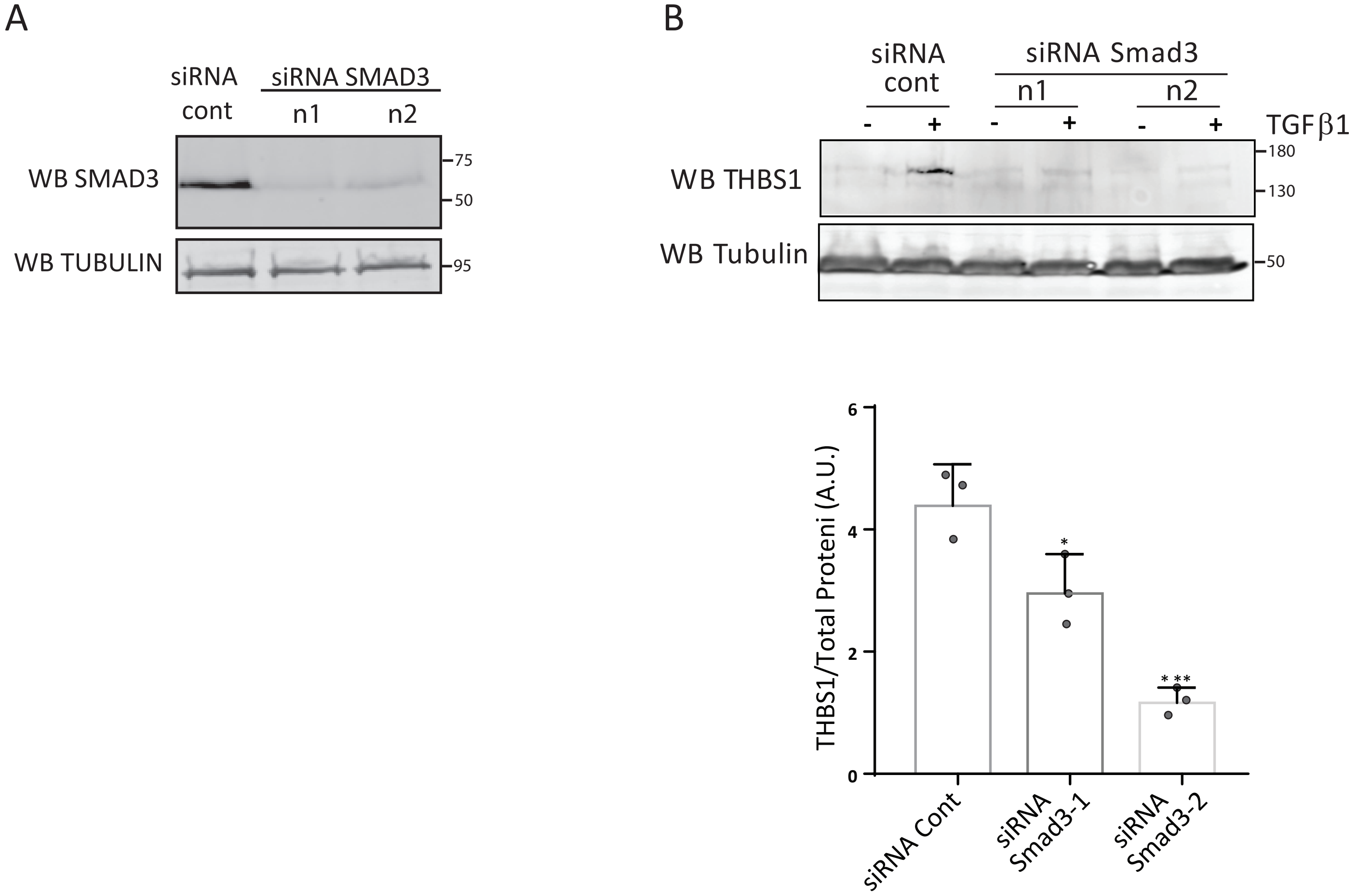

Supplementary figure 5 : Knock-down of SMAD3 interferes with THBS1 expression

(**A**) Immunoblots from protein extracts of non-transfected or transfected U87 cells with control siRNA (siRNA Cont) or SMAD3 siRNA (n1 or n2), and immunoblotted with anti-SMAD3 or anti-Tubulin antibodies.

(**B**) Immunoblots from protein extracts of U87 cells transfected with control siRNA (siRNA Cont) or SMAD3 siRNA (n1 or n2) treated or not with 5 ng/ml of recombinant TGFβ1 for 48 hours, and immunoblotted with anti-THBS1 or anti-Tubulin antibodies. The graph below represents a quantification of THBS1 signal over tubulin after TGFβ1 treatment . The results represent the means ± s.d. of three independent experiments. * $P< 0.05$; *** $P< 0.001$ (ANOVA, compared to control siRNA).

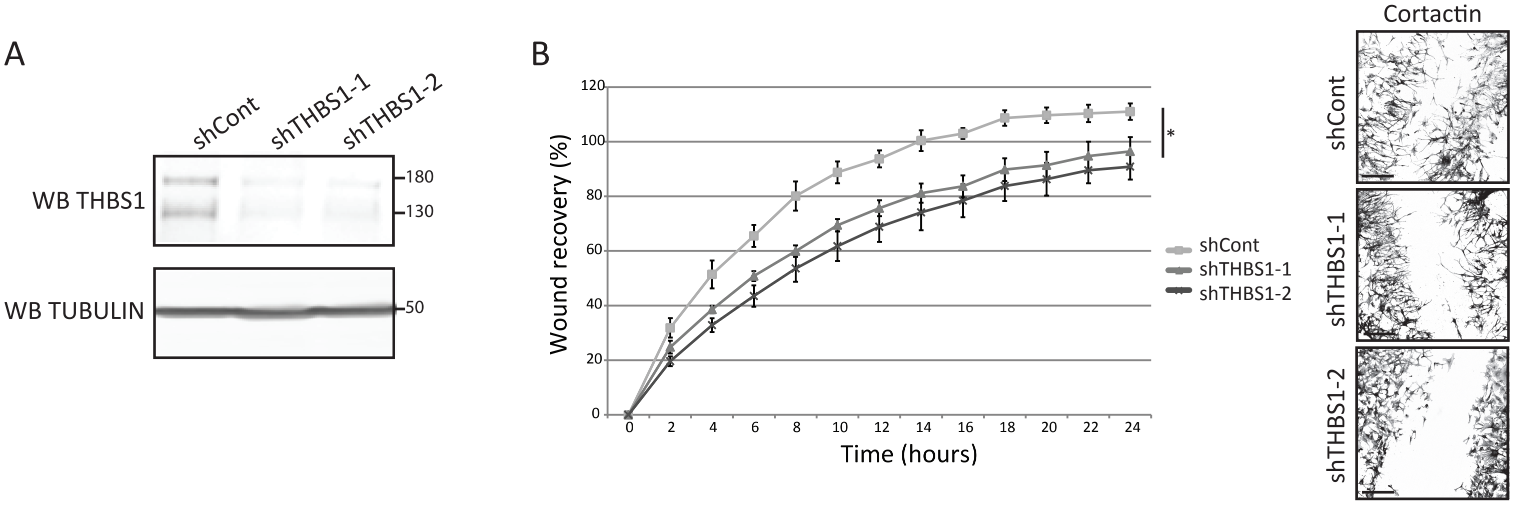

<u>Supplementary figure 6 </u>: THBS1 controls U87 cell invasion

(A) Immunoblots of protein extracts from U87 transfected cells with control (shCont) or THBS1 shRNAs (n1 or n2) probed with anti-THBS1 or anti-Tubulin antibodies.

(B) Invasion experiments of control or shTHBS1-1/-2 U87 cells in Matrigel. Cell invasion was automatically calculated by Odyssey® software every 2 hours, during 24 hours. The results represent the means ± s.d. of three independent experiments. * $P < 0.05$; ns, not significant (ANOVA). Representative images of U87 control or shTHBS1 cells immunostained with anti-Cortactin antibody (grey) are represented on the right panels. Scale bars: 20 µm.

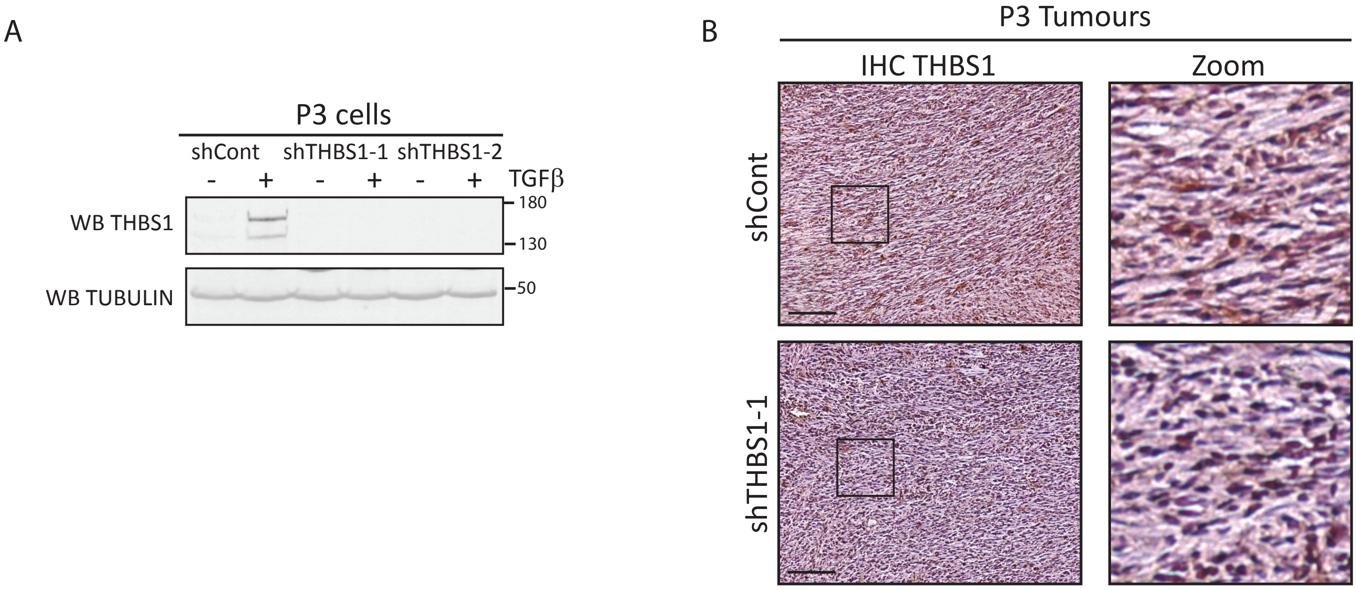

<u>Supplementary figure 7</u> : THBS1 knock-down in P3 cells and tumours

(**A**) Immunoblots of protein extracts from P3 transduced cells with control shRNA or THBS1-1/-2 shRNAs, treated for 48 hours with 10 ng/ml of TGFβ1 and probed with anti-THBS1 or anti-Tubulin antibodies.

(**B**) Representative images of control shRNA (upper panels) or THBS1-1 shRNA (lower panels) P3 tumours from mouse xenografts. Immunohistochemistry of THBS1 is represented in all panels (counterstaining with hematoxylin). Scale bars: 100 µm.

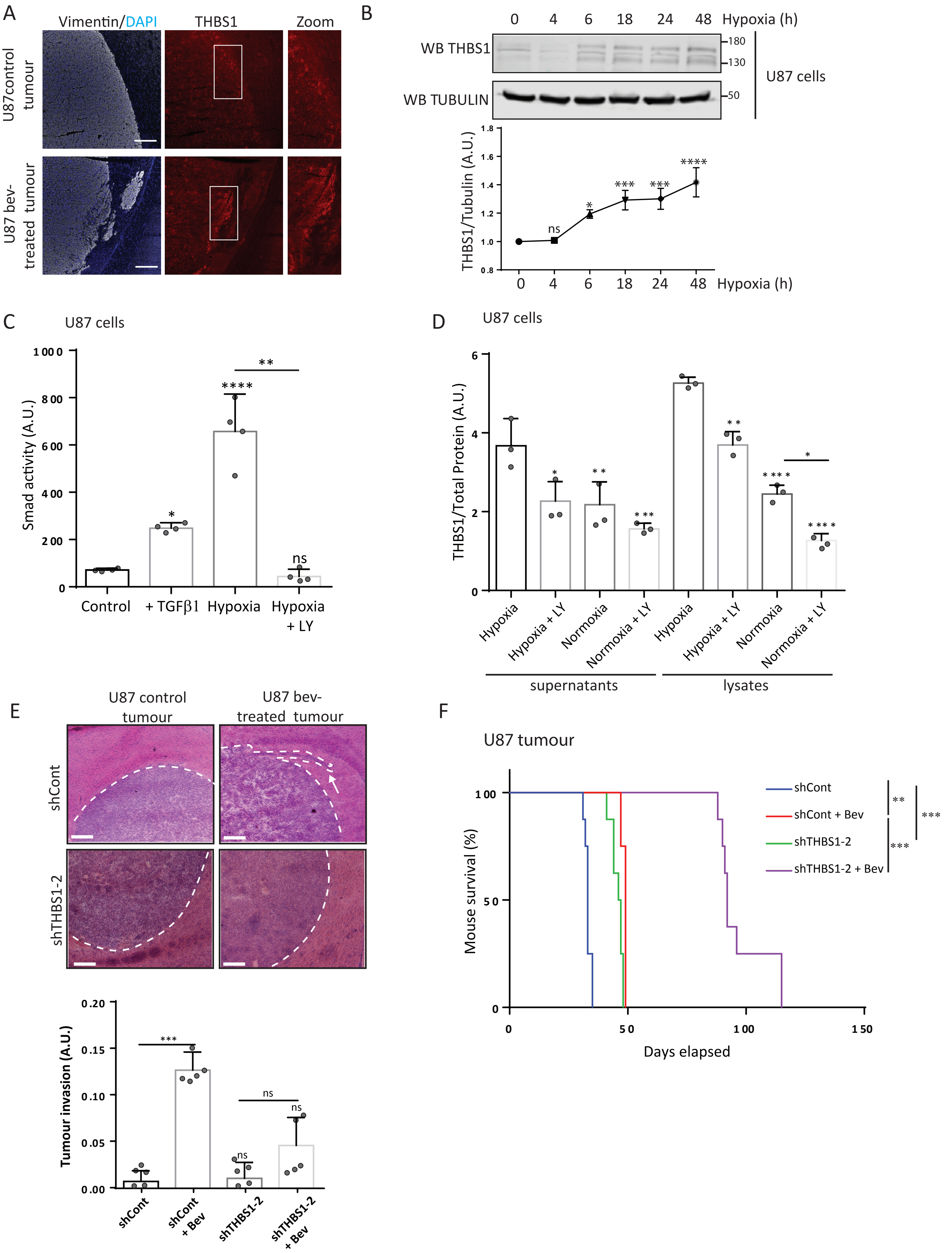

A
Vimentin/DAPI
THBS1
Zoom
U87control tumour
U87 bev-treated tumour
B
0 4 6 18 24 48 Hypoxia (h)
WB THBS1
WB TUBULIN
180
130
50
U87 cells
THBS1/Tubulin (A.U.)
1.8
1.6
1.4
1.2
1.0
0.8
ns
*
***
***
****
0 4 6 18 24 48 Hypoxia (h)
C
U87 cells
Smad activity (A.U.)
1000
800
600
400
200
0
**
****
*
ns
Control
+ TGFβ1
Hypoxia
Hypoxia + LY
D
U87 cells
THBS1/Total Protein (A.U.)
6
4
2
0
*
**
***
**
****
*
****
Hypoxia
Hypoxia + LY
Normoxia
Normoxia + LY
Hypoxia
Hypoxia + LY
Normoxia
Normoxia + LY
supernatants
lysates
E
U87 control tumour
U87 bev-treated tumour
shCont
shTHBS1-2
Tumour invasion (A.U.)
0.20
0.15
0.10
0.05
0.00
***
ns
ns
ns
shCont
shCont + Bev
shTHBS1-2
shTHBS1-2 + Bev
F
U87 tumour
Mouse survival (%)
100
50
0
0
50
100
150
Days elapsed
shCont
shCont + Bev
shTHBS1-2
shTHBS1-2 + Bev
**
***
***

<u>Supplementary figure 8</u>: THBS1 expression is increased by bevacizumab treatment and affects survival in mice

(A) Representative images of U87 control or bevacizumab-treated tumours. Tumours were immunostained with anti-THBS1 antibody (red) and anti-vimentin antibody (grey), and with DAPI (blue). THBS1 is increased after bevacizumab treatment. Scale bars: 100 µm.

(B) Immunoblots from protein extracts from U87 cells exposed to hypoxia (1% O_2) for 4, 6, 18 , 24 or 48 hours, and probed with anti-THBS1 or anti-Tubulin antibodies. The graph below represents ratios of THBS1 and tubulin signal intensities. The results are represented as means ± s.d. of three independent experiments.

(C) U87 cells were incubated under normoxia (21% O_2) or hypoxia (1% O_2) for 48 hours and stimulated or not with 5 ng/ml of recombinant TGFβ1 or 10µM of LY2157299. Cells were transfected with a SMAD Responsive Element construct and luciferase activity was assessed. The results are represented as means ± s.d. of four independent experiments.

(D) ELISA experiments performed on U87 cell lysates and supernatants. Cells were treated or not with 10 µM of TGFβR1 inhibitor LY2157299, in normoxic or hypoxic conditions for 48 hours. The graph represents three independent experiments as fold induction in comparison to non-treated cells (means ± s.d.).

(E) Representative images of U87 sh-control or shTHBS1-2 tumours treated or not with bevacizumab. Haematoxylin/Eosin staining reveals tumour borders (dashed lines) and invasive areas (arrow). Scale bars: 100 µm. The graph (below) depicts the results as the ratio of the surface of the invasive area to the total tumour surface in all conditions (means of 5 tumors ± s.d.). For all graphs : * $P< 0.05$; ** $P< 0.01$; *** $P< 0.001$; **** $P< 0.0001$; ns, non-significant (ANOVA).

(F) Kaplan-Meier survival curves of mice with intracranially implanted control (blue line), or THBS1-2 shRNA (green line) transduced U87 cells. Animals were treated or not with Bevacizumab (Bev). shCont + Bev (red line); shTHBS1-2 + Bev (purple). p values were calculated using a log-rank test (n=7 mice per group) ; ** p-value < 0.01 ; *** p-value < 0.001.

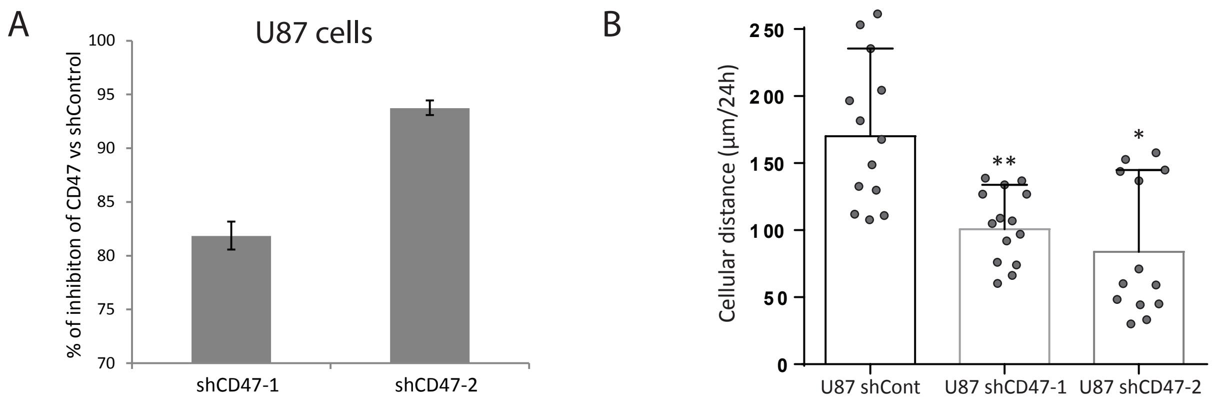

<u>Supplementary figure 9</u> : CD47 knock-down impairs U87 cell migration

(**A**) qPCR of CD47 transcripts in shCD47-1 or shCD47-2 transduced U87 cells in comparison with shControl transduced U87 cells.

(**B**) U87 cells were transduced with control or CD47 shRNAs (-1 and -2) and then tracked by videomicroscopy. The graph represents spontaneous migration recorded over 24 hours, as described in material and methods (means of 14 different fields ± s.d.). * $P< 0.05$; ** $P< 0.01$ (ANOVA).

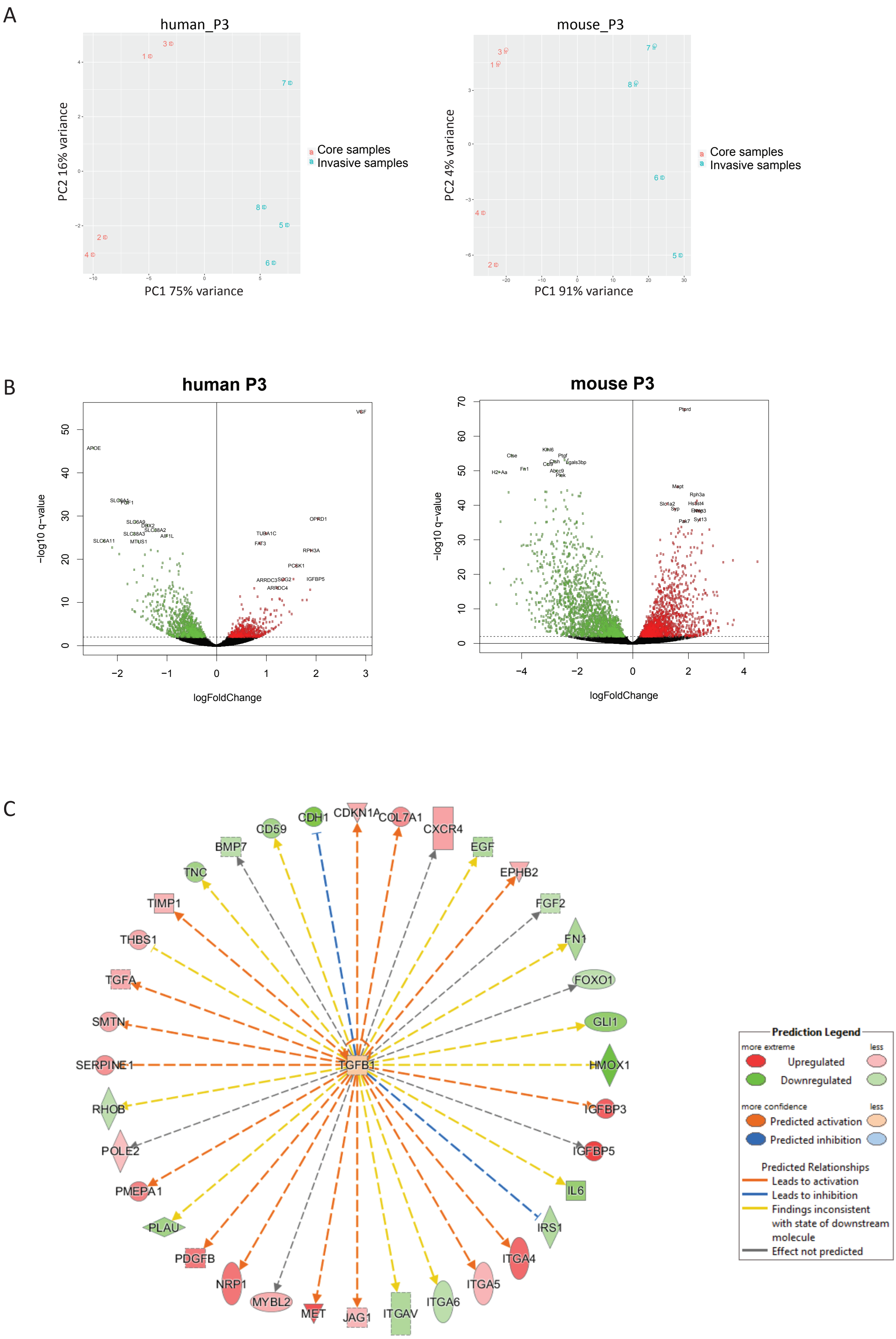
A
human_P3
PC1 75% variance
PC2 16% variance
Core samples
Invasive samples
mouse_P3
PC1 91% variance
PC2 4% variance
Core samples
Invasive samples
B
human P3
−log10 q−value
logFoldChange
APOE
VGF
OPRD1
TUBA1C
FAT3
RPH3A
PCSK1
IGFBP5
ARRDC3
ARRDC4
mouse P3
−log10 q−value
logFoldChange
Ptprd
Mapt
Rph3a
C
TGFB1
CD59
CDH1
CDKN1A
COL7A1
CXCR4
BMP7
EGF
TNC
EPHB2
TIMP1
FGF2
THBS1
FN1
TGFA
FOXO1
SMTN
GLI1
SERPINE1
HMOX1
RHOB
IGFBP3
POLE2
IGFBP5
PMEPA1
IL6
PLAU
IRS1
PDGFB
ITGA4
NRP1
ITGA5
MYBL2
MET
JAG1
ITGAV
ITGA6
Prediction Legend
more extreme
less
Upregulated
Downregulated
more confidence
less
Predicted activation
Predicted inhibition
Predicted Relationships
Leads to activation
Leads to inhibition
Findings inconsistent with state of downstream molecule
Effect not predicted

<u>Supplementary figure 10</u>: RNA sequencing analysis of core and invasive tumour areas

(A) Representations of the first and second component from a principle component analysis of mouse and human transcriptomes from core and invasive areas in P3 tumours. A large proportion of the variance is observed between the two groups (variation represented by separation on the x-axis: 91% for mouse, 75% for human).

(B) Volcano plot representations of mouse and human gene expression in P3 tumours. The red spots represent genes upregulated in invasive areas and the green spots represent genes upregulated in the tumour core.

(C) Interaction network (from Ingenuity Pathway Analysis) showing known gene-gene relationships involving TGFβ1 and genes differentially expressed between the tumour core and invasive areas. The ratio of expression of genes in the tumour core and invasive areas has been overlaid on to the network. Genes expressed higher in the core areas are green while genes expressed higher in invasive areas are pink. Arrows represent potential activation or inhibition by TGFβ1 of these targets, including THBS1.

Supplementary figure 11

<u>Uncropped blots from Figure 2A</u>

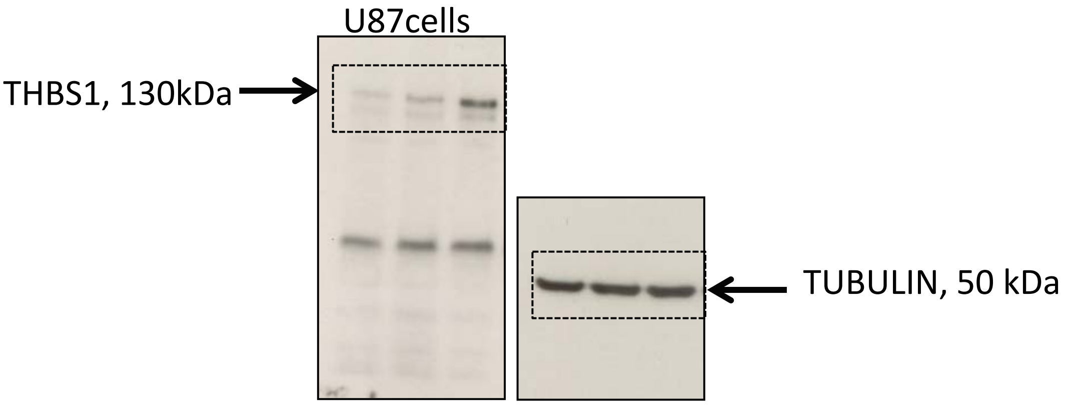

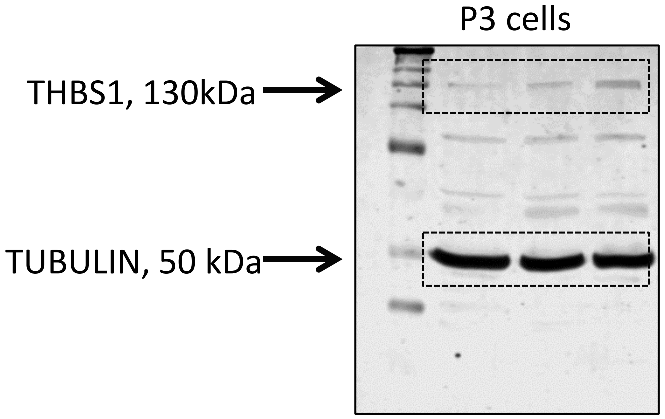

<u>Uncropped blots from Figure 3D</u>

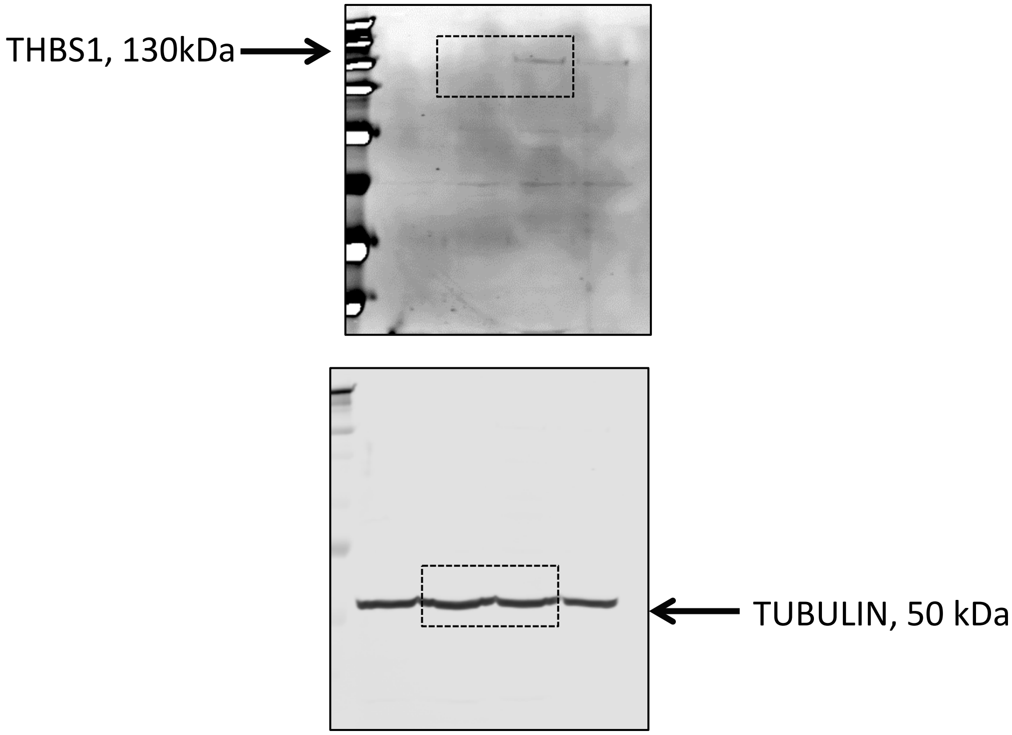

Supplementary figure 12

Uncropped blots from Figure 4A

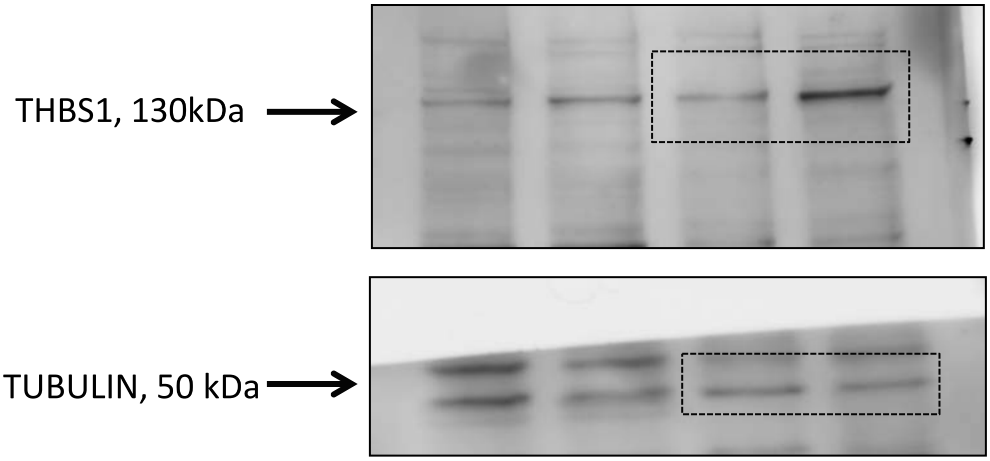

Uncropped blots from Figure 4C

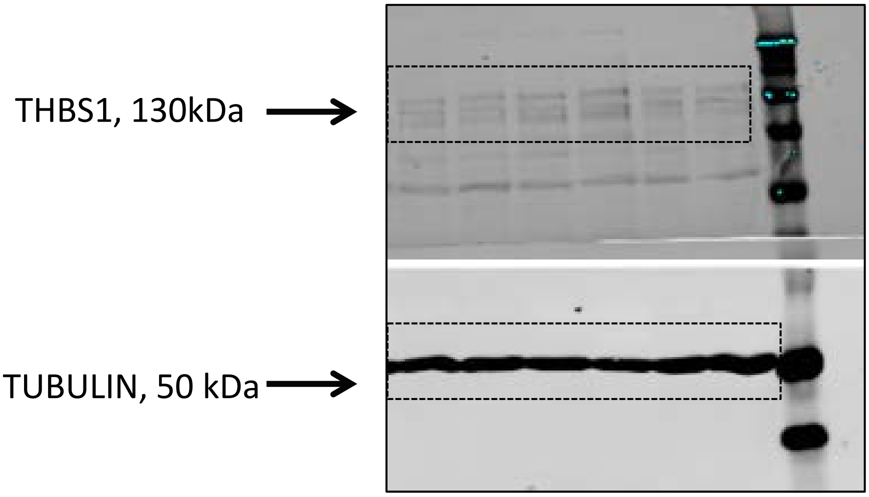

Uncropped blots from Figure 6A

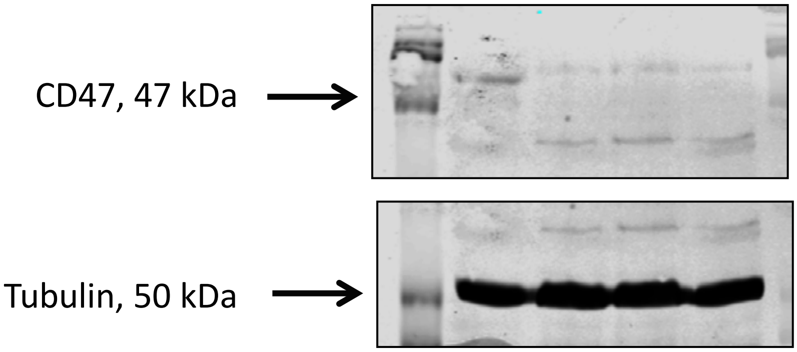

Supplementary Table 1. Patient tumour molecular profile (related to Supplementary Figure 2B)

	Molecular tumour profile
Patient 1	GBM IDH1 wt MGMT + (59%) MIB1:45%
Patient 2	GBM MIB18% IDH1 wt MGMT met
Patient 3	GBM IDH1wt MGMT met (42%) MIB1 10%
Patient 4	GBM IDH1 wt MGMT met (35%) MIB1 10%
Patient 5	GBM IDH1 wt MIB1 15% MGMT neg 1p+ 19q-
Patient 6	GBM IDH1 wt MGMT met (8%) MIB1 20%

Supplementary Table 2. Ingenuity Pathway Analysis (IPA) of P3 tumours

Gene	Category	P-value of overlap	Interactors within IPA database
TNF	cytokine	1.62E-07	ADAMTS4,ADGRG6,ALDH2,APOE,CAT,CD59,CDC42EP4,CDH1,CDKN1A,CXCR4,EPHB2,F2RL1,F3,FGFR2,FN1,HMOX1,IFIT3,IL17RB,IL6,ITGA4,ITGA5,ITGAV,LYN,NOS2,PLAU,PLSCR1,PPARA,PPARGC1A,PPP1R3C,PSEN1,RARRES3,RGS20,RND3,ROBO1,SEPP1,SERPINE1,SLC1A2,SOAT1,TGFA,TIMP1,TIMP3,TLR4,TM4SF1,TNC,TRAF1,VCAM1,WISP1
TGFB1	growth factor	3.25E-07	BMP7,CD59,CDH1,CDKN1A,COL7A1,CXCR4,EGF,EPHB2,FGF2,FN1,FOXO1,GLI1,HMOX1,IGFBP3,IGFBP5,IL6,IRS1,ITGA4,ITGA5,ITGA6,ITGAV,JAG1,MET,MYBL2,NRP1,PDGFB,PLAU,PMEPA1,POLE2,RHOB,SERPINE1,SMTN,TGFA,THBS1,TIMP1,TNC
PRL	cytokine	1.60E-06	CDH1,CDKN1A,CTSB,DDX58,GMPR,IFI44L,IFI6,IFIH1,IFIT1,IFIT3,IFIT5,IRF9,PLSCR1,SAMD9,SAMD9L
IFNL1	cytokine	3.59E-06	DDX58,IFI44L,IFI6,IFIH1,IFIT1,IFIT2,IFIT3,IFIT5,IL6,IRF9,PLSCR1,RARRES3,SAMD9
IGF1	growth factor	1.40E-05	CDKN1A,F3,FN1,GAP43,GFAP,HIF1A,IGFBP3,IRS1,ITGAV,ITGB5,PLAU,SERPINE1
IFNG	cytokine	2.25E-05	CASP1,CAT,CDKN1A,CELSR1,CXCR4,DDX58,FGF2,IFI44L,IFI6,IFIH1,IFIT1,IFIT3,IFIT5,IL17RB,IL18BP,IL6,IRF9,ITGAV,MCHR1,MTMR3,NOS2,PCSK1,PIM1,PLA2G16,RARRES3,SAMD9,SMTN,SOAT1,TGM1,TLR4,TRIL,VCAM1
IFNA2	cytokine	1.34E-04	DDX58,FGF2,HIF1A,IFI44L,IFI6,IFIH1,IFIT1,IFIT2,IFIT3,IFIT5,IL6,IRF9,PLSCR1
IL13	cytokine	3.52E-04	ABCA1,C3AR1,CASP1,CXCR4,GPNMB,HOMER2,IL13RA1,JAG1,JAKMIP2,MAOA,MN1,NOS2,PFKP,RIN2,SLC16A6,SLC26A6,SWAP70,THBS1,TIMP4,TRPS1
ITGA4	transmembrane receptor	4.08E-04	ITGA4,ITGA5,ITGAV
CTGF	growth factor	4.30E-04	ADAMTS4,ADAMTS5,FN1,SERPINE1,TIMP1,TIMP3

Supplementary Table 3. Connectivity map of P3 tumours (related to Figure 7)

Genes	Number of connections
THBS1	14
ANXA2	14
PDGFB	11
NRP1	9
IGFBP3	8
IGFBP5	8
LAMB1	8
SERPINE1	4

Supplementary Table 4. List of primary antibodies

Antibody	Manufacturer	Cat. No.	Working Conc/Dilution	Application	Host
AKT (11E7)	Cell Signaling	4685	1/10000	WB	Rabbit
CAIX (Carboxy Anhydrase IX)	Abcam	ab15086	1/500	IF	Rabbit
CD31	BD Pharmingen	553370	1/100	IF	Rat
CD47	BD Pharmingen	556044	1/500	WB	Mouse
Cortactin	Millipore	05-180	1/500	IF	Mouse
DAPI	Fisher	D1306	$1/10^6$	IF	
Endoglin (CD105)	BD Pharmingen	550546	1/50	IF	Rat
F-actin (I-19)	Santa Cruz	sc-1616	1/1000	IF	Goat
Fibronectin	Abcam	ab154211	1/1000	WB	Mouse
Fibronectin	Abcam	ab23750	1/600	IF	Rabbit
IRE1 α	Santa Cruz	sc-20790	1/1000	WB	Rabbit
Nestin (clone 10C2)	Millipore	MAB5326	1/500	IF	Mouse
pAKT (Ser473)	Cell Signaling	9271	1/1000	WB	Rabbit
pERK	Cell Signaling	9101	1/1000	WB	Rabbit
Phalloidin Rhodamine	Molecular Probes	R415	1/100	IF	
Psmad1-5 (S463/465) (41D10)	Cell Signaling	9516	1/500	WB	Rabbit
Psmad2 (S465/467)/Smad3 (S423/425) (D27F4)	Cell Signaling	8828	1/100	IF	Rabbit
Psmad2 (Ser465/467)	Cell Signaling	3108	1/500	WB	Rabbit
Psmad3	Abcam	ab52903	1/100	IF	Rabbit
Psmad3 (S423/425) (C25A9)	Cell Signaling	9520	1/500	WB	Rabbit
Smad1 (D59D7)	Cell Signaling	6944	1/500	WB	Rabbit
Smad2 (D43B4)	Cell Signaling	5339	1/500	WB	Rabbit
Smad3 (C67H9)	Cell Signaling	9523	1/500	WB	Rabbit
Smad4	Cell Signaling	9515	1/500	IF	Rabbit
TGFb1	Abcam	ab9758	1/200	WB	Rabbit
THBS1	Santa Cruz	59886	1/200	HIC	Mouse
THBS1	Home-made from JJ.Feige		1/500	IF	Rabbit
THBS1 (A6.1)	Thermo-Scientific	MA5-13398	1/500	WB	Mouse
Tubulin	Sigma	T5168	1/5000	WB	Mouse
Vimentin	Abcam	ab16700	1/200	IF	Rabbit
Vinculin	Sigma	V9131	1/500	WB	Mouse

Supplementary Table 5. List of secondary antibodies

ODYSSEY	Manufacturer	Cat. No.	Working Conc/Dilution
IRDye 800 donkey anti-Rabbit	Licor	926-32213	1/5000
IRDye 800 donkey anti-Mouse	Licor	926-32212	1/5000
IRDye 680 donkey anti-Rabbit	Licor	926-68023	1/5000
IRDye 680 donkey anti-Mouse	Licor	926-68022	1/5000
FLUORESCENCE	Manufacturer	Cat. No.	Working Conc/Dilution
Alexa Fluor 488 goat anti mouse IgG (H+L)	Invitrogen	A11001	1/500
Donkey Anti-Mouse IgG(H+L) 647H	Interchim	FP-SC4110	1/500
Alexa fluor 647 F(ab')2 fragment of goat anti Rabbit IgG(H+L)	Invitrogen	A-21246	1/500
Donkey Anti-Rabbit IgG(H+L) 488	Interchim	FP-SA5110	1/500
Alexa fluor 546 Goat anti rat IgG (H+L)	Invitrogen	A-11081	1/500
Alexa Fluor 546 donkey anti goat IgG (H+L)	Invitrogen	A11056	1/500

Supplementary Table 6. List of siRNA or shRNA with target sequences

siRNA Smad3	Ref J-020067-05 and J-020067-06	
shRNA hCD47	shRNA1	GCTACTGGCCTTGGTTTAATT
	shRNA2	TCCATGCTTTGTTACTAATAT
shRNA hTSP1	shRNA1	GCAACTACCTGGGCCACTATA
	shRNA2	TTGGAGATCAGTGTGACAATT
siRNA THBS1	ON-TARGET plus SMART Pool L-019743	GGACUGCGUUGGUGAUGUA
		CCAAAGACGGGUUUCAUUA
		CGAUGACUAUGCUGGAUUU
		CCACGAGGGCUCAGGGAUA

Supplementary Table 7. List of primers with sequences

hXBP1 Fwd	GGAGTTAAGACAGCGCTTGG
hXBP1 Rev	GAGATGTTCTGGAGGGGTGA
hTSP1 Fwd	TGCCTGATGACAAGTTCCAAG
hTSP1 Rev	CCAGAGTGGTCTTTCCGCTC
hCD47 Fwd	TCCGGTGGTATGGATGAGAAA
hCD47 Rev	ACCAAGGCCAGTAGCATTCTT
hbAct 1	CGTACCACTGGCATCGTGAT
hbAct 2	GTGTTGGCGTACAGGTCTTT
